# Is polycystic kidney disease associated with malignancy in children?

**DOI:** 10.1002/mgg3.725

**Published:** 2019-06-14

**Authors:** Brian D. Friend, Kami Wolfe Schneider, Timothy Garrington, Laurel Truscott, Julian A. Martinez‐Agosto, Robert S. Venick, Eileen Tsai Chambers, Patricia Weng, Douglas G. Farmer, Vivian Y. Chang, Noah Federman

**Affiliations:** ^1^ Department of Pediatrics UCLA Mattel Children's Hospital Los Angeles California; ^2^ Department of Pediatrics UCSF Benioff Children's Hospital San Francisco California; ^3^ Section of Hematology, Oncology, and Bone Marrow Transplantation University of Colorado Anschutz Medical Campus, Children's Hospital Colorado Aurora Colorado; ^4^ Department of Human Genetics UCLA David Geffen School of Medicine Los Angeles California; ^5^ UCLA Clinical Genomics Center Los Angeles California; ^6^ Division of Pediatric Nephrology, Department of Pediatrics Duke University Durham North Carolina; ^7^ Department of Surgery UCLA David Geffen School of Medicine Los Angeles California; ^8^ UCLA's Jonsson Comprehensive Cancer Center Los Angeles California; ^9^ Department of Orthopaedics UCLA David Geffen School of Medicine Los Angeles California

**Keywords:** genetic predisposition, genetic sequencing, malignancy, polycystic kidney disease

## Abstract

**Background:**

Polycystic kidney disease (PKD) is an inherited condition characterized by progressive development of end‐stage renal disease, hypertension, hepatic fibrosis, and cysts in the kidney, liver, pancreas, spleen, thyroid, and epididymis. While malignancies have been reported in association with PKD in adults, the incidence of malignancies in children with PKD is not currently known.

**Methods:**

We report on five patients with a known history of PKD who developed a malignancy as children at the University of California, Los Angeles and the University of Colorado Anschutz Medical Campus. Patients were included from 2012 to 2017.

**Results:**

We present five patients with a history of PKD diagnosed with a malignancy during childhood without any additional known mutations to suggest a genetic predisposition to develop cancer. This includes the first reported case of hepatocellular carcinoma in a patient with autosomal recessive polycystic kidney disease.

**Conclusion:**

Our report illustrates the potential that PKD may be associated with an increased risk for developing cancer, even in children. Further research is necessary to better understand this relationship.

## 1 INTRODUCTION

Polycystic kidney disease (PKD) is a heterogeneous disease with both autosomal dominant and recessive forms, each with distinct clinical characteristics. The recessive form, known as autosomal recessive polycystic kidney disease (ARPKD), results in end‐stage renal disease (ESRD) and congenital hepatic fibrosis in children (Hartung & Guay‐Woodford, 2014; MacRae Dell, 2011). In contrast, autosomal dominant polycystic kidney disease (ADPKD), typically presents during adulthood though symptoms may be observed in children and adolescents. The clinical course also results in ESRD as well as hypertension and cysts in several organs (MacRae Dell, 2011; Reddy & Chapman, 2017).

ADPKD results from pathogenic variants in the *PKD1* (OMIM *601313) and *PKD2* (OMIM *173910) genes located on chromosomes 4 and 16, respectively (Reddy & Chapman, 2017), while ARPKD is caused by mutations in the *PKHD1* (OMIM *606702) gene on chromosome 6 (Hartung & Guay‐Woodford, 2014). Other syndromes also can cause cysts in the kidneys such as tuberous sclerosis complex (TSC), von Hippel‐Lindau (VHL) disease, and neurofibromatosis type 1 (NF1), but these conditions are associated with other non‐cystic, characteristic features and have different molecular origins than ADPKD and ARPKD (Bonsib, 2009; Han & Criado, 2005).

While an increased prevalence of several malignancies has been reported in adults with PKD, few cases of children with PKD and pediatric malignancies have been described (Jilg et al., 2013; Yu et al., 2016). Reports as to whether a diagnosis of PKD is a risk factor for malignancy are conflicting. We present five pediatric patients with PKD who developed a malignancy during childhood, four of whom had histories of ADPKD, and one who represents the first reported case of hepatocellular carcinoma (HCC) diagnosed in a patient with ARPKD.

## 2 METHODS

### 2.1 Ethical compliance

This study was approved by the institutional review board at both institutions prior to data collection.

We describe five patients with a history of PKD that were diagnosed with cancer during childhood at the University of California, Los Angeles Medical Center and the University of Colorado Anschutz Medical Campus. Cases were included from 2012 to 2017 after examining the medical records of patients with PKD during this time period. In order to locate patients in the electronic medical record with PKD and cancer and to find relevant references in the literature, our search criteria included “polycystic kidney disease” OR “autosomal dominant polycystic kidney disease” OR “autosomal recessive polycystic kidney disease” OR “congenital multiple renal cysts” AND “cancer” OR “malignancy.”

## 3 RESULTS

### 3.1 ARPKD case report

A 5‐year‐old male with a history of kidney transplant secondary to ARPKD and associated congenital hepatic fibrosis was found to have biopsy‐proven HCC with pulmonary metastases. His liver mass initially measured 9.4 × 7.8 cm in the right upper lobe. He received neoadjuvant chemotherapy with cisplatin and doxorubicin, and had a partial response to therapy. This was followed by right liver lobectomy with negative tissue margins. The patient subsequently received seven cycles of adjuvant chemotherapy with vincristine, temozolamide, and irinotecan as well as thoracotomy to remove the remaining pulmonary nodules. His alpha‐fetoprotein decreased from 263,500 to 2.2 ng/ml after 14 months with ongoing evidence of complete remission by surveillance imaging. As of the latest follow‐up visit 17 months off‐therapy, the patient remains well and free of disease.

*PKHD1* gene sequencing identified two compound heterozygous pathogenic variants. One variant results in a frameshift mutation and a truncated protein that is expected to cause disease, but has not been previously reported in the literature (Table 1). The other variant is a substitution that causes an amino acid change, and has been described before in another individual with ARPKD (Krall et al., 2014).

**Table 1:** Characteristics of patients with a history of PKD who developed a malignancy

Patient	Age at cancer diagnosis	Gender	PKD type	PKD variant	Other genetic test results	Malignancy type
1	5 years	Male	ARPKD	*PKHD1* c.7967_7968delCA	No abnormalities identified by chromosomal microarray	Hepatocellular carcinoma
2	17 years	Male	ADPKD	*PKD1* c.1723−1G > A	No other variants identified by whole exome sequencing	Testicular germ cell tumor
3	15 years	Male	ADPKD	*PKD1* c.1723−1G > A	No other variants identified by whole exome sequencing	Testicular germ cell tumor
4	6 months	Female	ADPKD	Genetic testing not performed	Homozygous deletions of *SMARCB1* in tumor, both alleles present in blood	Renal rhabdoid tumor
5	8 years	Female	ADPKD	*PKD2* c.339delG	*TSC1*, *TSC2, PKD1* sequencing—no pathogenic variants detected	PEComa

Abbreviations: ADPKD, autosomal dominant polycystic kidney disease; ARPKD, autosomal recessive polycystic kidney disease; PEComa, perivascular epithelioid cell tumor; PKD, polycystic kidney disease; PKD1, polycystic kidney disease 1. GenBank Accession NM_001009944.2; PKD2, polycystic kidney disease 2. GenBank Accession NM_000297.3; PKHD1, polycystic kidney and hepatic disease 1. GenBank Accession NM_138694.3.

### 3.2 ADPKD case series

Four children with a history of ADPKD developed cancers during childhood. Two of these children (Cases 2 and 3) are siblings and were diagnosed with familial testicular germ cell tumors. The other two children with ADPKD were diagnosed with renal rhabdoid tumor (Case 4) and perivascular epithelioid cell tumor (PEComa) arising from the ligamentum teres with liver metastases (Case 5). At last follow‐up visit, all patients remain alive.

Case 2 and 3 were found to have the same mutation in *PKD1* that has been described previously (Truscott et al., 2017). Case 4 did not have genetic testing for PKD, but has a strong family history of ADPKD and a personal history of multiple renal cysts. Case 5 had genetic testing for TSC since PEComas and renal cysts have been reported in TSC (Dickson, Schwartz, Antonescu, Kwiatkowski, & Malinowska, 2013), however, sequencing and deletion/duplication analysis detected no mutations. Instead, a pathogenic variant in *PKD2* that resulted in a frameshift was detected. Further details are illustrated in Table 1.

### 3.3 Prevalence of cancer in children with PKD

Our search of the electronic medical records at our two centers identified 392 children with PKD from 2011 to 2015, and only five patients had a diagnosis of cancer. This translates to an incidence rate of 260 per 100,000 people with PKD per year.

## 4 DISCUSSION

In this report, we describe five patients with an underlying diagnosis of ADPKD or ARPKD who developed four different malignancy types during childhood. These patients had no other identified cancer predisposition syndromes, but did have confirmed diagnoses of PKD. This series also includes the first reported patient with ARPKD to develop HCC who had a novel pathogenic variant detected in the *PKHD1* gene. While the association of malignancy in PKD is unclear, patients' families were certainly impacted as they wondered if additional family members with this diagnosis might be at risk for cancer in the future.

Prior reports of malignancies in children with a history of PKD are limited. This includes a case of hepatoblastoma and pleuropulmonary blastoma that developed in two separate patients with a history of ARPKD (Luoto, Pakarinen, Jahnukainen, & Jalanko, 2014; Traubici, Somers, Ling, Pearl, & Nathan, 2011). Tumors associated with TSC have also been observed as TSC and PKD may occur together due to the concurrent deletion of both *PKD1* and *TSC2* (OMIM *191092) genes that are adjacent to chromosome 16 (Rijal, Dhakal, Giri, & Dahal, 2014). Surprisingly, although PEComas are known to be associated with TSC, no pathogenic variants in the *TSC1* (OMIM *605284) nor *TSC2* genes were detected in Case 5 (Dickson et al., 2013).

In contrast to the rare reports of malignancies in children diagnosed with PKD, solid tumors have been described in several studies in adults with PKD, suggesting an age‐related increased risk for malignancy. Mutations in the genes encoding polycystin‐1 and polycystin‐2 are responsible for ADPKD, and have been associated with oncogenesis (Gargalionis et al., 2015). One study that reviewed surgically removed kidneys in patients with a history of ADPKD found that 5% of the lesions were malignant (Jilg et al., 2013). Conversely, ARPKD is the result of mutations in *PKHD1*, whose protein polyductin is not known to increase the risk for malignancy, yet cases of cholangiocarcinoma have been reported in adults with ARPKD (Fonck, Chauveau, Gagnadoux, Pirson, & Grünfeld, 2001). The specific mutations described in this cohort have not been associated with cancer in other reported cases of PKD. More convincing, a large retrospective study found that there was an increased risk of several cancer subtypes in patients with PKD when compared to a cohort of patients without PKD (Yu et al., 2016). Two potential confounding factors that were not considered in the prior studies included a patient's history of renal dysfunction or solid organ transplantation, yet both have been associated with an increased risk of malignancy (Doycheva, Amer, & Watt, 2016; Russo, 2012). Among our patients, only case 1 should be mentioned as he had a kidney transplant nearly 2 years prior to his diagnosis of HCC.

These patients were all worked up for likely cancer predisposition syndromes, but no mutations were found to suggest a genetic predisposition in any of the cases. However, complete genetic profiling was not performed for any of our patients. ADPKD is relatively common in the adult population, and the estimated prevalence of solid tumors in this population appears to be higher than in the general population. While we cannot prove that ARPKD and ADPKD was the underlying cause of malignancy in our patients, given the rarity of the tumors especially in pediatrics, it seems possible that they may be associated with oncogenesis.

Further supporting our hypothesis is the high incidence of cancer that we have described in children with PKD at our two centers. Our data suggest an estimated incidence of 260 cases of cancer per 100,000 children with PKD per year, that is significantly higher than the incidence of childhood cancer (age 0–19 years) in the general population (18.2 per 100,000 per year) according to the most recent available SEER data (SEER, 2018). However, our estimate is likely greater than expected, given that ADPKD is frequently asymptomatic during childhood and therefore, it is possible that some children may have died from their malignancy without knowing that they also had a genetic diagnosis of PKD. In addition, a recent meta‐analysis revealed that ADPKD is likely an under‐recognized condition, confirming that while still rare, the diagnosis is often missed and is more common than previously reported (Solazzo et al., 2018). Therefore, while there are few reported cases of malignancy associated with PKD, the magnitude of cancer risk may be significant.

**Figure 1:**
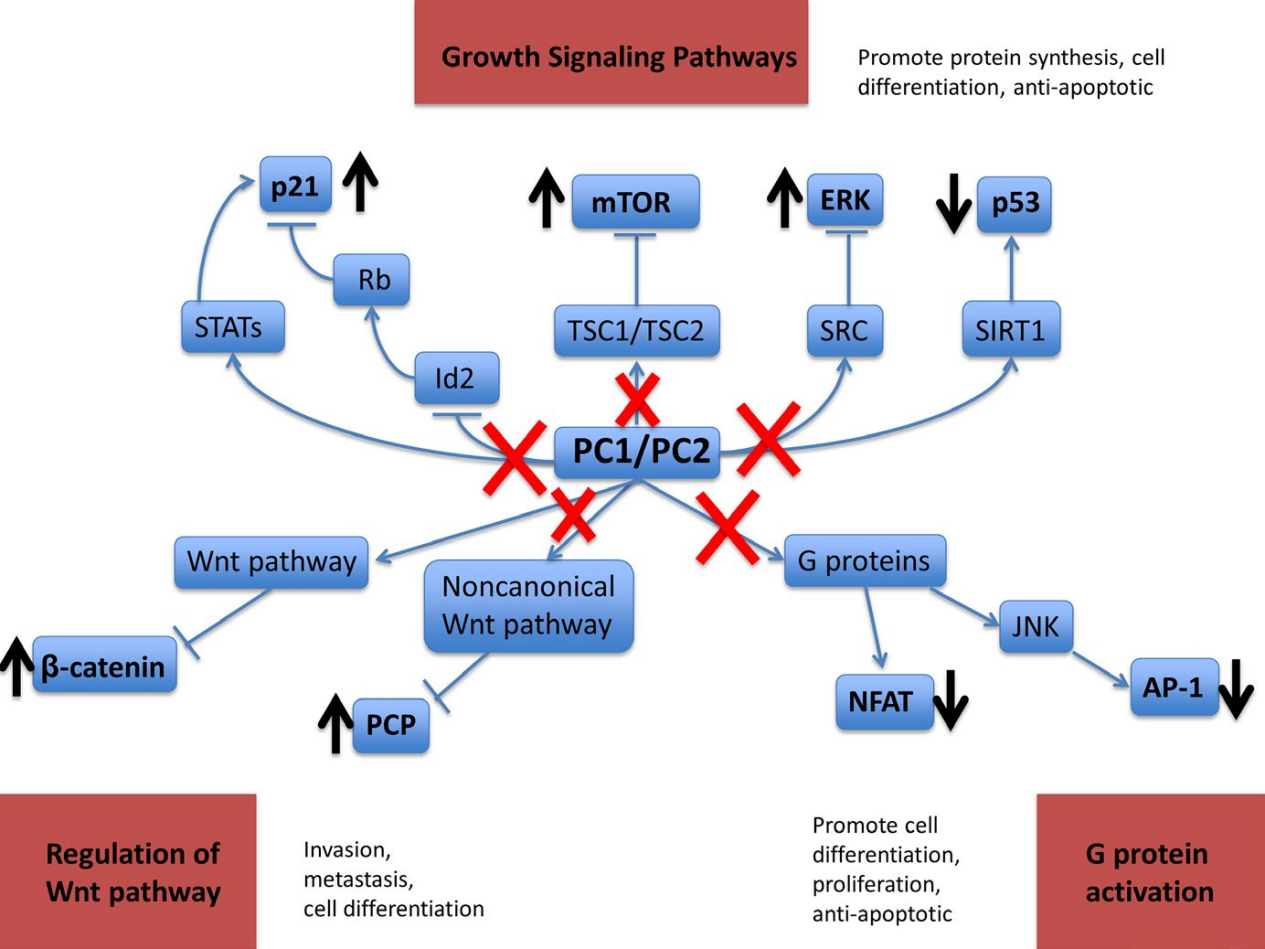
Signaling pathways present in the development of PKD that may promote oncogenesis (Chapin & Caplan, 2010; Nagao et al., 2012; Seeger‐Nukpezah et al., 2015)

Although the pathogenesis of PKD shares many similarities with malignant proliferation, some studies have shown that these cystic lesions are not associated with the development of malignancy, and may actually be protective (Orskov, Sørensen, Feldt‐Rasmussen, & Strandgaard, 2012; Seeger‐Nukpezah, Geynisman, Nikonova, Benzing, & Golemis, 2015; Wetmore et al., 2014). Yet, our reported cases suggest the possibility that the genetic mutations present in PKD may play a role in driving oncogenesis especially given the rarity of cancer in children. This seems most probable in ADPKD given the increased development of both cysts and malignancies in several other autosomal dominant cancer predisposition syndromes, for example, NF1, TSC, VHL (Crespigio et al., 2018; Kandt, 2003). All of these syndromes involve alterations of genes in the mammalian target of rapamycin (mTOR) pathway, which is known to regulate cell proliferation and differentiation. Moreover, polycystin‐1 has specifically been implicated in this pathway (Shillingford et al., 2006). Animal models of PKD have also shown upregulation and phosphorylation of cancer associate tyrosine kinases including AKT, ERK, B‐Raf, and Src (Nagao, Kugita, Yoshihara, & Yamaguchi, 2012). In Figure 1, we illustrate multiple genetic pathways including mTOR and several pathologic processes that have been described in cancer development and PKD. The overlap of these pathways suggests that patients with PKD may harbor alterations in the mTOR signaling pathways and have a higher risk for developing a malignancy than individuals in the general population.

Further research evaluating children with PKD prospectively and evaluating the incidence of cancer would potentially address this question. Such studies could include examining the prevalence of solid tumors in patients with ADPKD at our institutions in order to help determine the significance of our findings. If there truly is an increased risk to develop cancer in children with PKD, increased surveillance may be warranted to detect cancer at an early stage, or prevent tumors from becoming malignant. Additionally, gaining a better understanding of the molecular and biochemical mechanism of pediatric malignancy in the context of PKD could elucidate improved options for chemoprevention and/or treatment.

## CONFLICT OF INTEREST

The authors have no conflicts of interest to disclose.

## References

[mgg3725-bib-0001] Bonsib, S. M. (2009). Renal cystic diseases and renal neoplasms: A mini‐review. Clinical Journal of the American Society of Nephrology, 4, 1998–2007. 10.2215/CJN.02020309 19875768

[mgg3725-bib-0002] Chapin, H. C. , & Caplan, M. J. (2010). The cell biology of polycystic kidney disease. Journal of Cell Biology, 191, 701–710. 10.1083/jcb.201006173 21079243 PMC2983067

[mgg3725-bib-0003] Crespigio, J. , Berbel, L. C. L. , Dias, M. A. , Berbel, R. F. , Pereira, S. S. , Pignatelli, D. , & Mazzuco, T. L. (2018). Von Hippel‐Lindau disease: A single gene, several hereditary tumors. Journal of Endocrinological Investigation, 1, 21–31. 10.1007/s40618-017-0683-1 28589383

[mgg3725-bib-0004] Dickson, M. A. , Schwartz, G. K. , Antonescu, C. R. , Kwiatkowski, T. J. , & Malinowska, I. A. (2013). Extrarenal perivascular epithelioid cell tumors (PEComas) respond to mTOR inhibition: Clinical and molecular correlates. International Journal of Cancer, 132, 1711–1717. 10.1002/ijc.27800 22927055 PMC3558545

[mgg3725-bib-0005] Doycheva, I. , Amer, S. , & Watt, K. D. (2016). *De novo* malignancies after transplantation: Risk and surveillance strategies. Medical Clinics of North America, 100, 551–567. 10.1016/j.mcna.2016.01.006 27095645

[mgg3725-bib-0006] Fonck, C. , Chauveau, D. , Gagnadoux, M. F. , Pirson, Y. , & Grünfeld, J. P. (2001). Autosomal recessive polycystic kidney disease in adulthood. Nephrology Dialysis Transplantation, 16, 1648–1652. 10.1093/ndt/16.8.1648 11477168

[mgg3725-bib-0007] Gargalionis, A. N. , Korkolopoulou, P. , Farmaki, E. , Piperi, C. , Dalagiorgou, G. , Adamopoulos, C. , … Papavassiliou, A. G. (2015). Polycystin‐1 and polycystin‐2 are involved in the acquisition of aggressive phenotypes in colorectal cancer. International Journal of Cancer, 136, 1515–1527. 10.1002/ijc.29140 25123959

[mgg3725-bib-0008] Han, M. , & Criado, E. (2005). Renal artery stenosis and aneurysms associated with neurofibromatosis. Journal of Vascular Surgery, 41, 539–543. 10.1016/j.jvs.2004.12.021 15838492

[mgg3725-bib-0009] Hartung, E. A. , & Guay‐Woodford, L. M. (2014). Autosomal recessive polycystic kidney disease: A hepatorenal fibrocystic disorder with pleiotropic effects. Pediatrics, 134, e833–845. 10.1542/peds.2013-3646 25113295 PMC4143997

[mgg3725-bib-0010] Jilg, C. A. , Drendel, V. , Bacher, J. , Pisarski, P. , Neeff, H. , Drognitz, O. , … Neumann, H. P. H. (2013). Autosomal dominant polycystic kidney disease: Prevalence of renal neoplasias in surgical kidney specimens. Nephron Clinical Practice, 123, 13–21. 10.1159/000351049 23752029

[mgg3725-bib-0011] Kandt, R. S. (2003). Tuberous sclerosis complex and neurofibromatosis type 1: The two most common neurocutaneous diseases. Neurologic Clinics, 21, 983–1004. 10.1016/S0733-8619(03)00004-5 14743661

[mgg3725-bib-0012] Krall, P. , Pineda, C. , Ruiz, P. , Ejarque, L. , Vendrell, T. , Camacho, J. A. , … Ars, E. (2014). Cost‐effective PKHD1 genetic testing for autosomal recessive polycystic kidney disease. Pediatric Nephrology, 29, 223–234. 10.1007/s00467-013-2657-7 24162162

[mgg3725-bib-0013] Luoto, T. T. , Pakarinen, M. P. , Jahnukainen, T. , & Jalanko, H. (2014). Liver disease in autosomal recessive polycystic kidney disease: Clinical characteristics and management in relation to renal failure. Journal of Pediatric Gastroenterology and Nutrition, 59, 190–196. 10.1097/MPG.0000000000000422 24806835

[mgg3725-bib-0014] MacRae Dell, K. (2011). The spectrum of polycystic kidney disease in children. Advances in Chronic Kidney Disease, 18, 339–347. 10.1053/j.ackd.2011.05.001 21896375 PMC3168776

[mgg3725-bib-0015] Nagao, S. , Kugita, M. , Yoshihara, D. , & Yamaguchi, T. (2012). Animal models for human polycystic kidney disease. Experimental Animals, 61, 477–488. 10.1538/expanim.61.477 23095811

[mgg3725-bib-0016] Orskov, B. , Sørensen, V. R. , Feldt‐Rasmussen, B. , & Strandgaard, S. (2012). Changes in causes of death and risk of cancer in Danish patients with autosomal dominant polycystic kidney disease and end‐stage renal disease. Nephrology Dialysis Transplantation, 27, 1607–1613. 10.1093/ndt/gfr467 21873624

[mgg3725-bib-0017] Reddy, B. V. , & Chapman, A. B. (2017). The spectrum of autosomal dominant polycystic kidney disease in children and adolescents. Pediatric Nephrology, 32, 31–42. 10.1007/s00467-016-3364-y 27034070

[mgg3725-bib-0018] Rijal, J. P. , Dhakal, P. , Giri, S. , & Dahal, K. V. (2014). Tuberous sclerosis complex with autosomal dominant polycystic kidney disease: A rare duo. BMJ Case Reports, 2014, 2014 10.1136/bcr-2014-207471 PMC4275747 25519866

[mgg3725-bib-0019] Russo, P. (2012). End stage and chronic kidney disease: Associations with renal cancer. Frontiers in Oncology, 2, 28 10.3389/fonc.2012.00028 22649783 PMC3355889

[mgg3725-bib-0020] Seeger‐Nukpezah, T. , Geynisman, D. M. , Nikonova, A. S. , Benzing, T. , & Golemis, E. A. (2015). The hallmarks of cancer: Relevance to the pathogenesis of polycystic kidney disease. Nature Reviews Nephrology, 11, 515–534. 10.1038/nrneph.2015.46 25870008 PMC5902186

[mgg3725-bib-0021] Shillingford, J. M. , Murcia, N. S. , Larson, C. H. , Low, S. H. , Hedgepeth, R. , Brown, N. , … Weimbs, T. (2006). The mTOR pathway is regulated by polycystin‐1, and its inhibition reverses renal cystogenesis in polycystic kidney disease. Proceedings of the National Academy of Sciences of the United States of America, 103, 5466–5471. 10.1073/pnas.0509694103 16567633 PMC1459378

[mgg3725-bib-0022] Solazzo, A. , Testa, F. , Giovanella, S. , Busutti, M. , Furci, L. , Carrera, P. , … Magistroni, R. (2018). The prevalence of autosomal dominant polycystic kidney disease (ADPKD): A meta‐analysis of European literature and prevalence evaluation in the Italian province of Modena suggest that ADPKD is a rare and underdiagnosed condition. PLoS ONE, 13, e0190430 10.1371/journal.pone.0190430 29338003 PMC5770025

[mgg3725-bib-0023] Surveillance, Epidemiology, and End Results Program . (2018). SEER Cancer Statistics Review, 1975‐2015. Table 28.1. Bethesda, MD: National Cancer Institute Retrieved from https://seer.cancer.gov/csr/1975_2015/browse_csr.php?sectionSEL=28&pageSEL=sect_28_table.01

[mgg3725-bib-0024] Traubici, J. , Somers, G. R. , Ling, S. C. , Pearl, R. J. , & Nathan, P. C. (2011). Pleuropulmonary blastoma in a child with autosomal‐recessive polycystic kidney disease. Pediatric Radiology., 41, 1465–1468. 10.1007/s00247-011-2026-2 21720858

[mgg3725-bib-0025] Truscott, L. , Gell, J. , Chang, V. Y. , Lee, H. , Strom, S. P. , Pillai, R. , … Federman, N. (2017). Novel association of familial testicular germ cell tumor and autosomal dominant polycystic kidney disease with PKD1 mutation. Pediatric Blood & Cancer, 64, 100–102. 10.1002/pbc.26197 27577987 PMC5937546

[mgg3725-bib-0026] Wetmore, J. B. , Calvet, J. P. , Yu, A. S. , Lynch, C. F. , Wang, C. J. , Kasiske, B. L. , & Engels, E. A. (2014). Polycystic kidney disease and cancer after renal transplantation. Journal of the American Society of Nephrology, 25, 2335–2341. 10.1681/ASN.2013101122 24854270 PMC4178444

[mgg3725-bib-0027] Yu, T.‐M. , Chuang, Y.‐W. , Yu, M.‐C. , Chen, C.‐H. , Yang, C.‐K. , Huang, S.‐T. , … Kao, C.‐H. (2016). Risk of cancer in patients with polycystic kidney disease: A propensity‐score matched analysis of a nationwide, population‐based cohort study. The Lancet Oncology, 17, 1419–1425. 10.1016/S1470-2045(16)30250-9 27550645

